# Case Report: First Case of Endophthalmitis Caused by an Emerging Pathogen: *Nocardia huaxiensis*

**DOI:** 10.3389/fpubh.2022.933851

**Published:** 2022-07-14

**Authors:** Chang Liu, Lei Zhang, Lili Liu, Ye Wang, Yanhui Cui, Tianwei Liang, Tianming Chen, Yongqiang Jiang, Gang Liu, Li Li

**Affiliations:** ^1^The Department of Ophthalmology, Beijing Children's Hospital Affiliated With Capital Medical University, Beijing, China; ^2^State Key Laboratory of Pathogen and Biosecurity, Beijing Institute of Microbiology and Epidemiology, Academy of Military Medical Sciences (AMMS), Beijing, China; ^3^The Department of Infectious Disease, Beijing Children's Hospital Affiliated With Capital Medical University, Beijing, China

**Keywords:** endophthalmitis, nocardiosis, antibiotic susceptibility, Nanopore sequencing, pathogen identification

## Abstract

*Nocardia* endophthalmitis is a relatively uncommon form of endophthalmitis seen in clinical patients. In general, *Nocardia* endophthalmitis tends to carry a poor prognosis. Here, we report a 3-year-old child who was admitted to the hospital due to a rupture of the left eye. The suturing and anterior chamber formation were performed immediately. Approximately, 16 days after the operation, massive whitish plump and tufted exudates gathered in the pupil area and at the bottom of the anterior chamber, and the child was diagnosed with endophthalmitis. The infection was initially considered to be caused by fungal pathogens for that the hyphae and spores were observed in the smear. However, the isolate obtained after 4 days of culturation was identified as actinomycetes using MALDI–TOF. We further classified it as *Nocardia huaxiensis* by next-generation sequencing (NGS) based on the MinION platform. Amikacin and sulfamethoxazole tablets were used to control the infection and the ocular inflammation subsided gradually. Intraocular lens (IOL) implantation is planned to be performed at an appropriate future time to improve his vision. *Nocardia* endophthalmitis is rare and usually caused by ocular trauma or surgery. In conclusion, *Nocardia huaxiensis* should be considered as an emerging pathogen and deserves more attention.

## Introduction

*Nocardia* species are a kind of aerobic, gram-positive, and weakly acid-fast bacteria that are commonly found in soil, water, and plants (1). In the past few years, an increasing number of species have been recognized as human pathogens and they are frequently associated with pulmonary infections, mycetoma, and disseminated nocardiosis (2, 3). They can also cause ocular morbidities such as keratitis, scleritis, and endophthalmitis (2). *Nocardia* had been reported to be associated with all the types of endophthalmitis, including post-operative, post-traumatic, and endogenous endophthalmitis, with generally poor-visual outcomes (4).

In recent years, the taxonomy of *Nocardia* has become more complex due to the description of new species that have made some clusters bigger or have even led to the creation of new ones (5). Species-level identification of *Nocardia* relies heavily on biochemical tests and cellular fatty acid analysis, which are cumbersome, ime-consuming, and often not definitive (6). In total, 16S rRNA gene sequencing was considered to be the “gold standard” for *Nocardia* species identification (7). Multi-locus sequence typing (MLST) using concatenated sequences of 5 housekeeping genes (*gyrB, 16S, secA, hsp65*, and *rpoB*) was also used to provide higher accuracy and discriminatory power in species identification of the *Nocardia* genus (6, 8). However, some reports have confirmed that the 16S rRNA sequence or the MLST method cannot provide enough genetic information to distinguish between closely-related *Nocardia* species (6, 9). A recent study showed that the *dapb1* gene, which encodes dipeptidyl aminopeptidase BI, was far superior to commonly-used markers for *Nocardia* and yielded a topology almost identical to that of whole genome-based phylogeny (9).

*Nocardia huaxiensis* (*N.huaxiensis*) was first isolated from skin biopsy specimens of a patient and identified as a novel *Nocardia* species in 2021 (10). There was no other reported case of human infection caused by this species. Next-generation sequencing (NGS) has the potential to determine pathogen species more specifically and accurately (11). In this report, we present a case of bacterial endophthalmitis caused by *N. huaxiensis* which was mistaken for fungal endophthalmitis before the correct diagnosis was made with NGS testing based on MinION platform. We also performed gram-staining and weak acid-fast staining (modified Kinyoun's method) of this strain and its susceptibility to antibiotics was tested using a broth dilution method according to Clinical and Laboratory Standards Institute (CLSI) standard M24-A2 guidelines. These results show that *N. huaxiensis* should be considered as an emerging pathogen and deserves more attention.

## Case Presentation

**Figure 1:**
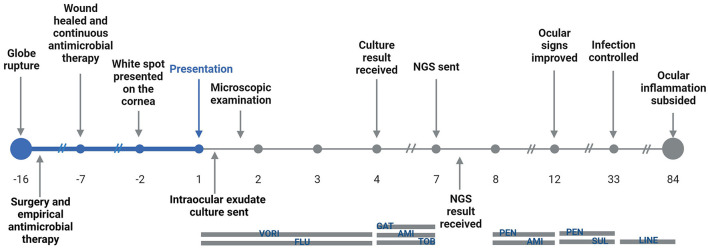
Timeline of the illness progress, pathogen identification, and treatment for the patient. VORI, voriconazole; FLU, fluconazole; GAT, gatifloxacin; AMI, amikacin; TOB, tobramycin; PEN, penicillin; SUL, sulfamethoxazole; LINE, linezolid.

A 3-year-old was admitted to the Emergency Department of Beijing Children's Hospital, Capital Medical University with photophobia and a white spot on the cornea of the left eye that had been presented for 2 days. The timeline is shown in Figure 1 and the complete case progress record is reported later in detail.

Approximately, 16 days before admission, the child fell on the garden steps, resulting in a globe rupture of the left eye with lacerations of the cornea and the sclera. The iris incarcerated in the wound. Corneal and scleral laceration debridement, suturing, and anterior chamber formation were performed immediately. To prevent post-traumatic endophthalmitis, empirical antimicrobial therapy was performed during the pre- and post-operative period with intravenous cefuroxime for 5 days and anti-inflammatory dexamethasone for 3 days post-operatively. In addition, levofloxacin antibiotic eye drops, tobramycin dexamethasone eye ointment, and prednisone acetate eye drops were administered for further anti-inflammatory and anti-infection purposes. In total, 7 days before admission, the corneal wound healed, and the suture remained tight. The anterior chamber was clear with medium depth, and the lens was also clear. Topical antibiotics were continued, and amblyopia treatment was initiated.

**Figure 2:**
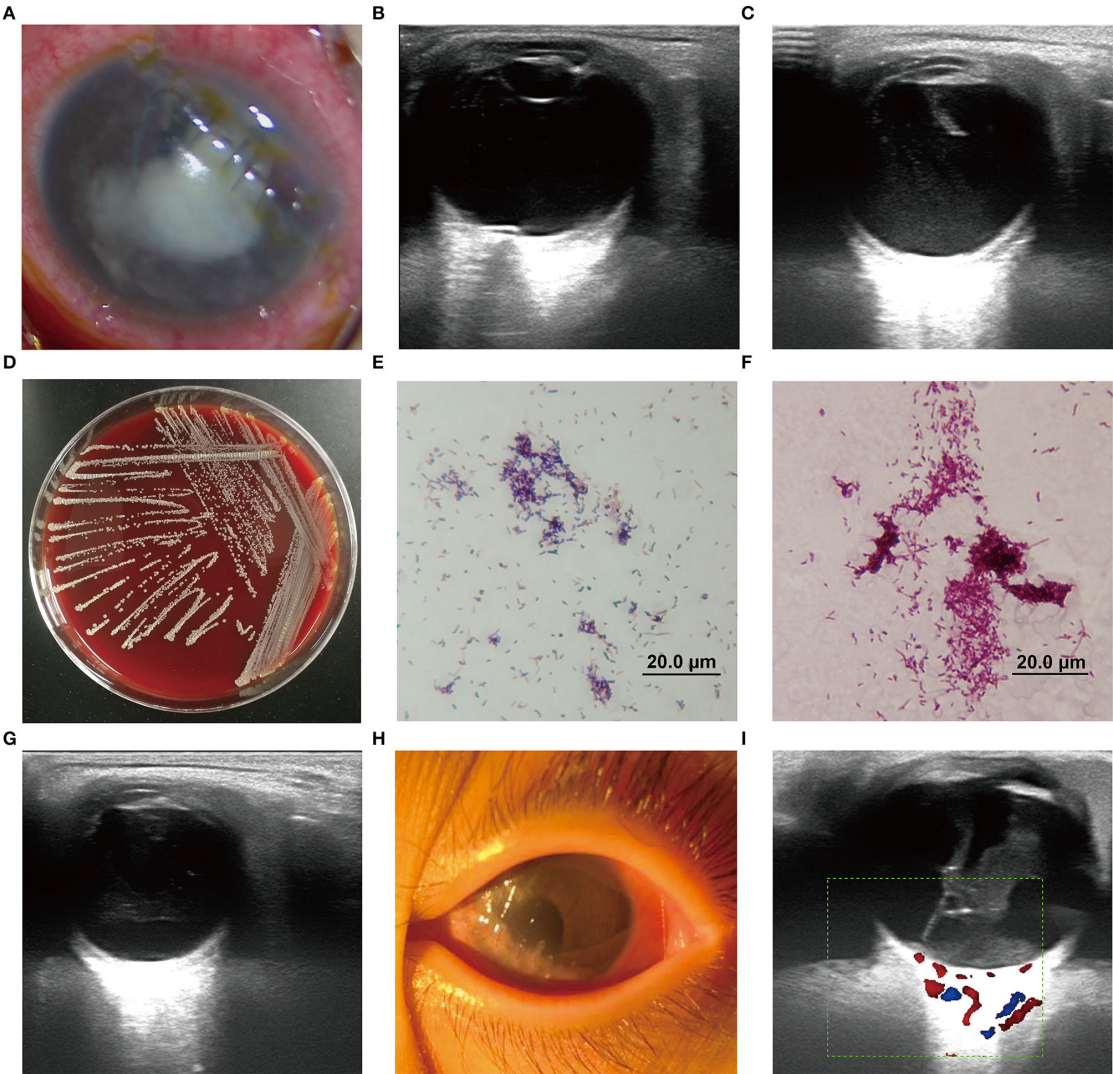
**(A)** Left eye with a massive white exudate gathered in the pupil area. **(B,C,G,H)** B-mode ultrasound of the left eye in different treatment stages. **(D)** The culture colonies of strain BCHNH01^T^ on blood agar plate after 48 h culture. **(E,F)** The gram-staining and weak acid-fast staining (modified the Kinyoun's method) of strain BCHNH01^T^. **(I)** Vitreoretinal traction and suspicious retinal detachment were observed in the left eye.

On admission, the patient was observed with mixed hyperemia and mild edema of the cornea as well as a shallow anterior chamber in the left eye. A massive white exudate gathered in the pupillary area and at the bottom of the anterior chamber. The iris was partially visible and the lens was completely invisible. Red light reflection was not observed (Figure 2A). The vital signs were stable. A systemic work-up revealed no obvious abnormalities in the respiratory system, digestive system, or nervous system. The indexes of liver and kidney function and chest X-ray were normal. B-mode ocular ultrasound showed an abnormal echo in the left eyeball and slight vitreous opacity (Figure 2B), suggestive of endophthalmitis caused by microbial infection. Emergency surgery was performed immediately, including anterior chamber irrigation, extracapsular cataract extraction (ECCE), and intravitreal drug injection in the left eye. During the surgery, the intraocular exudate was rinsed and sent for smear examination and culture (using sabouraud medium and columbia blood agar media, under aerobic conditions at 25 and 35°C, respectively). The lens cortex was sucked out, because opacity of the capsular membrane was observed. The optic disc appeared to be red, and the fundus was sightless. Intravitreal injection of ceftazidime (1 mg, 0.1 ml), vancomycin (1 mg, 0.1 ml), and also subconjunctival injection of tobramycin (20,000 U, 0.5 ml) were conducted. Adjunctive antimicrobial therapy was started after surgery with levofloxacin eye drops and gatifloxacin eye gel.

Microscopic examination revealed hyphae and spores, thus, the endophthalmitis was originally considered to be caused by fungal agents. Based on this finding, antimicrobial treatment was modified to 135 mg intravenous infusion of voriconazole combined with voriconazole eye drops every hour and subconjunctival injection of fluconazole. Intravitreal injection of voriconazole (0.1 mg, 0.1 ml) was given to the patient on the following day after surgery. On hospital day 3, ocular B-mode ultrasound showed an abnormal echo in the left eyeball and vitreous opacity (Figure 2C). Meanwhile, corneal edema, shallow anterior chamber, and aqueous flare were still present, indicating that the condition had not improved. On hospital day 4, the medias at 35°C showed that some yellow, dry, and chalky colonies were present after 48 h of culturation (Figure 2D) and were identified as actinomycetes by MALDI–TOF mass spectrometry. Following the recommendation of the Department of Infectious Diseases, administration with gatifloxacin eye gel, amikacin eye drops, and 5 consecutive days of subconjunctival injection of tobramycin were initiated to enhance anti-infection effects. Vitrectomy and intravitreal injection [ceftazidime (1 mg, 0.05 ml) and amikacin (0.4 mg, 0.1 ml)] were performed on hospital day 6.

On hospital day 7, in order to determine the pathogen species of the colonies accurately, the isolate was further subjected to whole-genome sequencing (WGS) on a MinION platform with Rapid Barcoding Kit (SQK-RBK004, Oxford Nanopore Technologies, ONT), as described in Supplementary Methods. Within 2 h, the MinION-based NGS assay determined that the species belonged to *Nocardia* spp. Subsequently, gram stain and weak acid-fast stain were performed and both were positive in the isolates (Figures 2E,F). Thus, we adjusted the treatment strategy again. Systemic use of penicillin and amikacin drugs and compound sulfamethoxazole tablets (0.48 g oral) were given as anti-infection agents. Amikacin eye drops, sulfacetamide sodium and penicillin eye drops, gatifloxacin eye gel, and conjunctival injection of penicillin were given per day.

On hospital day 12, the ocular congestion and the corneal edema had subsided, and the anterior chamber was gradually cleared. However, the ocular B-ultrasound indicated that the vitreous opacity in the left eye had not significantly changed (Figure 2G). In this period, to find out if the severe endophthalmitis aroused the intracranial infection, we took cranial MRI for him and no obvious abnormality was observed in bilateral brain parenchyma. Penicillin and sulfamethoxazole oral compound were continuously used for anti-infection. Amikacin was replaced with ceftriaxone sodium from experience due to the high risk of hearing loss in children with its long-term use. Through the continuous application of the above anti-infection treatment for 3 weeks, the infection was effectively controlled: eye congestion was reduced; the cornea became transparent; the anterior chamber was quiet with no cell or flare being noted; and the purulent secretion disappeared (Figure 2H). Thereafter, the doses were tapered as the infection was brought under control. Treatment with systemic intravenous cephalosporins and penicillin antibiotics was stopped, and linezolid was given orally. On hospital day 34, vitreoretinal traction and suspicious retinal detachment were observed (Figure 2I). Vitrectomy was recommended to relieve vitreoretinal traction. To further treat the vitreoretinal traction, the child underwent vitrectomy on the left eye.

On hospital day 84, the ocular inflammation subsided and the ocular structure was stable. No systemic complications were observed. The vital indexes were always steady during the treatment and no medication side effect appeared. Intraocular lens (IOL) implantation was planned to be performed at an appropriate time to improve vision.

## Discussion

Clinically, nocardiosis is a rare and potentially life-threatening gram-positive bacterial infection. The route of infection is usually *via* inhalation or direct inoculation (12). Commonly reported human infections with *Nocardia* are pulmonary nocardiosis, cutaneous nocardiosis, and brain abscesses (13, 14), while there are also some reports about endophthalmitis caused by *Nocardia* (4). *Nocardia* endophthalmitis is considered of a relatively rare but potentially devastating ocular condition (15). *N. huaxiensis* is a novel species identified in 2021, and here, we present the first description of endophthalmitis caused by this species.

Nocardiosis typically affects immunocompromised patients while this report describes an immunocompetent child who suffered ocular trauma by the steps in their garden yard. Our first potential speculation was that the infectious agent penetrated the eye during the primary injury and was possibly encapsulated around the injury to the anterior lens capsule. Our second potential speculation was the treatment time of glucocorticoids was too long. In addition to broad-spectrum antibiotics, the patient was also treated with glucocorticoids during the post-traumatic period, similarly to that reported by Compte et al. (16). *Nocardia* endophthalmitis is rare and these two reasons may work together to result in eye infection. Ocular exposure to soil or plant matter was a common historical point in case of *Nocardia* infection in eyes (17, 18) and our report also indicates that *Nocardia* infection should be considered in patients with such plant-inflicted trauma.

Clinical diagnosis of *Nocardia* is very difficult and complicated, as there are no signs, symptoms, or radiological findings that are pathognomonic for *Nocardia* infection (12). As such, clinical *Nocardia* endophthalmitis misdiagnosed as fungal endophthalmitis have been reported (19). Recently, MALDI–TOF has been shown to provide accurate identification of *Nocardia* species (8). However, while some species are easily identified, the identification of uncommon species remains a challenge (8). In this report, we mistook this case as a fungal infection due to the hyphae and spores observed in the smear. The colonies were observed after 4 days of culture and identified as actinomycetes *via* MALDI–TOF, while we could not identify the bacteria on species level. Nanopore sequencing is an emerging NGS technique that can generate sequencing reads in real time (20). During the sequencing of the pure culture isolate, the first reads file was outputted in 10 min after run started. Following analysis of the sequences determined it as the *Nocardia* genus initially, indicating that 10-mininute sequencing time was sufficient to enable pathogen identification by MinION. We reclassified this case as *Nocardia* Endophthalmitis and the amikacin treatment was used to cure the patient, which indicated that Nanopore sequencing can provide valuable information during the identification of novel *Nocardia* infections.

**Figure 3:**
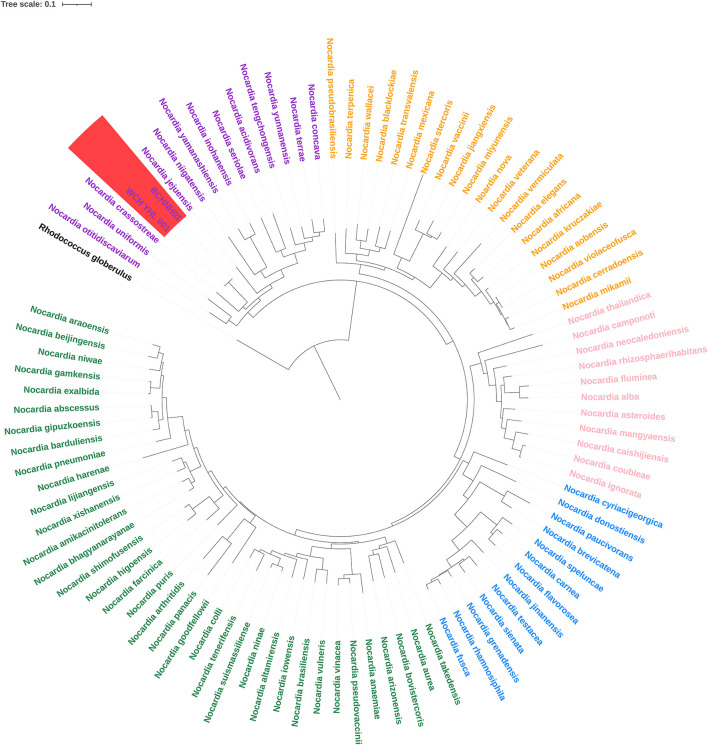
Phylogenomic tree was constructed based on the *dapb1* gene.

The whole genome of this isolate was assembled using Nanopore sequencing and the Illumina Noveseq platform. The genome size of this strain is 8.3 M nucleotides with a G+C content of 67%. We extracted the whole 16S rRNA sequence from the assemble genome and fount it showed the highest similarities (99.93%) with the 16S rRNA sequence of the strain *Nocardia huaxiensis* WCH-YHL-001^T^. We further compared the Average Nucleotide Identity (ANI) of the two strains and found that the ANI value was determined of 99.47%, indicating that the strain belong to a novel *Nocardia* species, *N. huaxiensis*, whose phylogenetic analysis was performed based on the 16S rRNA sequence (10). However, some reports have confirmed that the 16S rRNA sequence cannot provide enough genetic information to distinguish between closely-related species (8). The *dapb1* gene of *Nocardia* has been recently demonstrated to yield a topology almost identical to the genome-based phylogeny (9). The whole-genome sequences of 95 *Nocardia* strains were downloaded from the National Center for Biotechnology Information (NCBI) and the sequences of the *dapb1* gene were extracted. Multiple sequence alignment was performed with ClustalW2 and a phylogenetic tree was constructed using the maximum likelihood method with 1,000 bootstrap repeats with IQ–TREE. *Rhodococcus globerulus* NBRC 14531^T^ served as an outgroup. The phylogenomic tree revealed five main phylogroups, consistent with the previous report (9), and the strains BCHNH01^T^ and WCH-YHL-001^T^ formed independent branches with robust bootstrap support, indicating that they indeed belong to a new species (Figure 3).

Because of the variety of antibiotic susceptibility depending on *Nocardia* species, it is important to identify *Nocardia* at the species level and to investigate its antibiotic susceptibility (21). Amikacin at a concentration of 2–2.5% is considered to be the best choice for the treatment of *Nocardia* endophthalmitis, as amikacin demonstrates the lowest minimum inhibitory concentrations for *Nocardia* isolates (4). However, some cases of amikacin-resistant *Nocardia* have been described (22, 23), so it is important to test the antibiotic susceptibility of the *N. huaxiensis*. We performed the antimicrobial susceptibility of this strain by using a broth dilution method according to the CLSI standard M24-A2 guidelines. The strain was resistant to amoxicillin-clavulanic acid, ceftriaxone, imipenem, cefepime, and cefotaxime (Table 1). Interestingly, these drugs collectively belong to β-lactams which indicates that β-lactam antibiotics are not suitable to cure infection caused by this species.

**Table 1:** The antibiotic susceptibility of *Nocardia huaxiensis*.

**Antibiotics**	**Minimum inhibitory concentration (MIC, μg/mL)**	**Interpretation**
Amikacin	2	S
Amoxicillin-Clavulanic acid	≥64/32	R
Ceftriaxone	≥128	R
Ciprofloxacin	0.5	S
Clarithromycin	4	I
Imipenem	≥32	R
Linezolid	2	S
Minocycline	1	S
Moxifloxacin	1	S
Trimethoprim-Sulfamethoxazole	1/19	S
Tobramycin	4	S
Cefepime	≥64	R
Cefotaxime	≥128	R
Doxycycline	0.5	S

*S, Susceptible; R, Resistant; I, Intermediate*.

A limitation of this case study is that we did not identify *N. huaxiensis* from the intraocular exudate directly. Although we identified the cultured isolate correctly with Nanopore sequencing, we wasted nearly 1 week while waiting for the isolate to grow. Nanopore sequencing has the advantage of rapid library preparation and real-time data acquisition and it has been used to identify viral and bacterial pathogens from clinical samples directly (24). Whether the Nanopore sequencing can be used in the rapid species-level identification of the *Nocardia* genus from the clinical samples deserves more investigation. Besides, we did not test the antimicrobial susceptibility of this strain in a timely manner, which led to the improper use of some antibiotics such as ceftriaxone.

This case study demonstrates that it is of great importance to explore new pathogen identification strategy to improve the prognosis of the patients even though the treatment process is tortuous. Accurate identification of *Nocardia* species, complete local debridement, and appropriate antibiotic therapy are important in the treatment of *Nocardia* infections. At present, the child is still being treated with local eye drops combined with systemic oral drugs to control infection. However, the effects of long-term treatment with anti-infection agents still need to be observed moving forward.

In conclusion, here, we described a case of bacteria endophthalmitis caused by *N. huaxiensis*, which was mistakenly diagnosed as fungal endophthalmitis. *Nocardia* endophthalmitis is often difficult to diagnose, mimicking chronic inflammation, or fungal infection. Ophthalmologists should be aware of infections caused by *Nocardia* and suspect *Nocardia* endophthalmitis after plant-inflicted trauma. Earlier and accurate identification of *Nocardia* species can improve patient outcomes. We have initially revealed the application prospects of Nanopore sequencing in the identification of *Nocardia* infection, especially for novel and uncommon species. The antibiotic susceptibility of *N. huaxiensis* was also tested according to CLSI standard M24-A2 guidelines and β-lactam antibiotics were found to be unsuitable to cure the infection caused by this species. *N. huaxiensis* is an emerging pathogen and we hope that our results can provide some instructions for future treatment.

## Data Availability Statement

The datasets for this article are not publicly available due to concerns regarding participant/patient anonymity. Requests to access the datasets should be directed to the corresponding author.

## Ethics Statement

The studies involving human participants were reviewed and approved by the Ethics Committee of Beijing Children's Hospital Affiliated with Capital Medical University. Written informed consent to participate in this study was provided by the participants' legal guardian/next of kin. Written informed consent was obtained from the individual(s), and minor(s)' legal guardian/next of kin, for the publication of any potentially identifiable images or data included in this article.

## Author Contributions

CL analyzed and interpreted patient data. LZ performed the Nanopore sequencing and antimicrobial susceptibility test experiment. LLiu performed microscopic examinations of this strain and performed the surgery described in the case report with YC, TL, and TC. YW analyzed data and improved the manuscript in details. YJ and GL performed the literature review and wrote the manuscript. LLi conceived the idea of describing the case report and reviewed, and made significant revisions to this manuscript. All authors read and approved the final manuscript.

## Funding

This project was financed by the National Natural Science Foundation of China (82002115 and 81772144).

## Conflict of Interest

The authors declare that the research was conducted in the absence of any commercial or financial relationships that could be construed as a potential conflict of interest.

## Publisher's Note

All claims expressed in this article are solely those of the authors and do not necessarily represent those of their affiliated organizations, or those of the publisher, the editors and the reviewers. Any product that may be evaluated in this article, or claim that may be made by its manufacturer, is not guaranteed or endorsed by the publisher.
