# Supplementary material for: Case Report: First Case of Endophthalmitis Caused by an Emerging Pathogen: Nocardia huaxiensis
Source: Front Public Health. 2022 Jul 14;10:933851. doi: 10.3389/fpubh.2022.933851 (PMC9330429; doi:10.3389/fpubh.2022.933851)
Supplement: Supplementary file 1 [file Presentation_1.PDF]

Whole genome sequencing (WGS) based on MinION

DNA extraction

DNA of the *Nocardia huaxiensis* isolate was extracted using IndiSpin Pathogen Kit (Indical Bioscience) according to the manufacturer's protocol.

Library preparation and sequencing

1. Prepare the NEBNext Ultra II End Repair/dA-Tailing Module reagents according to the manufacturer's instructions, and place on ice.
2. Mix the following reagents in a 0.2 mL thin-walled PCR tube:

**Component**	**Volume**
Near 100 ng template DNA	50 µL
Ultra II End-prep reaction buffer	7 µL
Ultra II End-prep enzyme mix	3 µL
**Total**	**60 µL**

3. Mix well by gently pipetting the entire volume within the tube up and down 10 times.
4. Using a thermal cycler, incubate at 20°C for 5 mins and 65°C for 5 mins.
5. Resuspend the AMPure XP beads by vortexing.
6. Transfer the sample to a clean 1.5 mL Eppendorf DNA LoBind tube.
7. Perform Agencourt AMPure XP beads purification with 60 µL beads and elute with 16 µL Nuclease-free water.
8. Thaw the Blunt/TA Ligase Master Mix, spin down and mix by pipetting the entire volume within the tube up and down 10 times. Check for any precipitate (if any is visible, continue to mix) and place on ice.
9. Thaw the Barcode Adapter (BCA), spin down and mix by pipetting the entire volume within the tube up and down 10 times. Place on ice.
10. Add the reagents in the order given below, mixing by flicking the tube between each sequential addition:

**Component**	**Volume**
End-prepped DNA	15 µL
Barcode Adapters (BCA)	10 µL
Blunt/TA Ligase Master Mix	25 µL
**Total**	**50 µL**

11. Mix well by gently pipetting the entire volume within the tube up and down 10 times.
12. Incubate the reaction for 10 minutes at RT.

13. Perform Agencourt AMPure XP beads purification with 30 μL beads and elute with 25 μl Nuclease-free water.
14. Quantify 1 μL of adapted DNA using a Qubit fluorometer.
15. Calculate how much DNA to take forward into the PCR step for a final DNA concentration of 0.2 ng/μL in a 50 μL reaction.
16. Thaw the LongAmp® Hot Start Taq 2X Master Mix at RT, spin down and mix by pipetting the entire volume within the tube up and down 10 times. Place on ice.
17. Thaw the required Barcode Primers at RT, spin down and mix by pipetting the entire volume within the tube up and down 10 times. Place on ice.
18. Set up the adapted DNA PCR as follows:

**Component**	**Volume**
Adapter ligated DNA	diluted x μL 0.2 ng/μL
Barcode Primers (BP01-12, at 10 μM)	1 μL
LongAmp® Hot Start Taq 2x Master Mix	25 μL
Nuclease-free water	24-x μL
**Total**	**50 μL**

19. Mix well by gently pipetting the entire volume within the tube up and down 10 times.
20. Amplify using the following cycling conditions:
    1) Initial denaturation 3 mins @ 95 °C (1 cycle)
    2) Denaturation 15 secs @ 95 °C (15 cycles)
    3) Annealing 15 secs @ 56 °C (15 cycles)
    4) Extension 2 min @ 65 °C (15 cycles)
    5) Final extension 6 mins @ 65 °C (1 cycle)
    6) Hold @ 4 °C
21. Perform Agencourt AMPure XP beads purification with 50 μL beads and elute with 10μL 10 mM Tris-HCl (pH 8.0 with 50mM NaCl).
22. Pool all barcoded libraries in the desired ratios to a total of 50-100 fmoles in 10 μL of 10 mM Tris-HCl (pH 8.0 with 50 mM NaCl).
23. Perform the rapid adapter (RAP) ligation and priming and loading the SpotON flow cell according to the universal protocol.
24. Wash the flow cell with SQK-WSH004 kit according to the universal washing protocol.

Agencourt AMPure XP beads purification (Universal)

1. Resuspend the AMPure XP beads for use by vortexing about 30min in advance.
2. Add **appropriate volume** of resuspended AMPure XP beads to the reaction and mix by pipetting.
3. Incubate on a Hula mixer (rotator mixer) for 5 minutes at RT.

4. Prepare 5mL of fresh 70% ethanol in Nuclease-free water.
5. Spin down the sample and pellet on a magnet. Keep the tube on the magnet, and pipette off the supernatant.
6. Keep the tube on the magnet and wash the beads with 200 µL of freshly prepared 70% ethanol without disturbing the pellet. Remove the ethanol using a pipette and discard.
7. Repeat the previous step.
8. Spin down and place the tube back on the magnet. Pipette off any residual ethanol. Allow to dry for ~30 seconds, but do not dry the pellet to the point of cracking.
9. Remove the tube from the magnetic rack and resuspend pellet with **appropriate volume** 10 mM Tris-HCl (pH 8.0 with 50mM NaCl) or nuclease-free water. Incubate for 5 minutes at RT.
10. Pellet the beads on a magnet until the eluate is clear and colorless.
11. Remove and retain all of eluate into a clean 1.5 mL Eppendorf DNA LoBind tube.

Rapid adapter (RAP) ligation and loading library preparation (Universal)

1. Add 1 µL of RAP to the barcoded DNA.
2. Mix gently by flicking the tube, and spin down.
3. Incubate the reaction for 5 minutes at room temperature (RT).
4. Mix the following reagents in a 0.2 mL thin-walled PCR tube:

Component	Volume
Sequencing Buffer (SQB)	34 µL
Loading beads (LB)	25.5 µL
Nuclease-free water	4.5 µL
**Total**	**75 µL**

5. Priming and loading the SpotON flow cell.

Bioinformatics pipeline

The sequencing reads were generated by MinIT with Guppy software (version 4.3.4, Oxford Nanopore Technologies) via real time base-calling. The barcodes and adapters were trimmed using Guppy with the command "guppy-barcoder". Following demultiplexing, Minimap2 (version 2.17) was used to computationally subtract host reads, with the "-ax map-ont" setting, by aligning reads to the human reference genome (GRCH38, https://ftp.ncbi.nlm.nih.gov/genomes/all/GCF/000/001/405/GCF_000001405.39_GRCh38.p13/). All remaining non-human reads were separated by SAMtools (version 1.7) and the FASTQ file outputs were converted to FASTA files with SeqKit (version 0.13.2). FASTA reads were then mapped to the RefSeq bacterial database (containing 2328 bacterial genomes or scaffolds, https://ftp.ncbi.nlm.nih.gov/genomes/refseq/) by BLASTn (version 2.10.1). To make

the results after BLASTn more intuitive, two python scripts were developed and performed, which are available in https://github.com/gitzl222/mNPS/.
